# Inhibition of West Nile Virus by Calbindin-D28k

**DOI:** 10.1371/journal.pone.0106535

**Published:** 2014-09-02

**Authors:** Venkatraman Siddharthan, Hong Wang, Christopher J. Davies, Jeffery O. Hall, John D. Morrey

**Affiliations:** 1 Institute for Antiviral Research, Department of Animal, Dairy, and Veterinary Sciences, Utah State University, Logan, Utah, United States of America; 2 Center for Integrated BioSystems, Department of Animal, Dairy, and Veterinary Sciences, Utah State University, Logan, Utah, United States of America; 3 Utah Veterinary Diagnostic Laboratory, Department of Animal, Dairy, and Veterinary Sciences, Utah State University, Logan, Utah, United States of America; University of Hong Kong, Hong Kong

## Abstract

Evidence indicates that West Nile virus (WNV) employs Ca^2+^ influx for its replication. Moreover, calcium buffer proteins, such as calbindin D28k (CB-D28k), may play an important role mitigating cellular destruction due to disease processes, and more specifically, in some neurological diseases. We addressed the hypothesis that CB-D28k inhibits WNV replication in cell culture and infected rodents. WNV envelope immunoreactivity (ir) was not readily co-localized with CB-D28k ir in WNV-infected Vero 76 or motor neuron-like NSC34 cells that were either stably or transiently transfected with plasmids coding for CB-D28k gene. This was confirmed in cultured cells fixed on glass coverslips and by flow cytometry. Moreover, WNV infectious titers were reduced in CB-D28k-transfected cells. As in cell culture studies, WNV env ir was not co-localized with CB-D28k ir in the cortex of an infected WNV hamster, or in the hippocampus of an infected mouse. Motor neurons in the spinal cord typically do not express CB-D28k and are susceptible to WNV infection. Yet, CB-D28k was detected in the surviving motor neurons after the initial phase of WNV infection in hamsters. These data suggested that induction of CB-D28k elicit a neuroprotective response to WNV infection.

## Introduction

Calcium (Ca^2+^) plays a pivotal cellular role in signal transduction pathways in nearly all cell processes. Intracellular Ca^2+^ is tightly regulated by the integrity of membranes, regulated ion channels, and controlled calcium exchange between mainly the extracellular milieu, endoplasmic reticulum, and sarcoplasmic reticulum [1], [2]. As such, cells need to achieve Ca^2+^ homeostasis while still maintaining a >10,000-fold gradient across plasma membranes.

Ca^2+^ has also been found to play a role in almost every step of virus replication cycles, depending on the virus. Ca^2+^ can play roles in calcium-dependent enzymatic processes, mitochondrial boosting of ATP production to achieve higher energy demands, inhibiting protein trafficking pathways via the endoplasmic reticulum and Golgi to prevent immune reactions, and induction or prevention of apoptosis through modulation of ER-mitochondria Ca^2+^ coupling (reviewed [2]).

In regard to the effect of calcium on West Nile virus (WNV) infection, a study by Scherbik and Brinton [3] demonstrates that infection leads to cytosolic Ca^2+^ influx in different types of cultured cells. The virus employs Ca^2+^ influx for its replication, probably by activating cellular processes favoring viral replication. This influx also results in early caspase-3 cleavage. Inhibitors of Ca^2+^ influx at early times of infection decrease viral yield by >2 log_10_, decreases caspase-3 cleavage, and activate putative cell-protective kinases, which extends cell survival.

Evidence suggests that calcium buffer proteins may play an important role mitigating cellular destruction due to disease processes, and more specifically, in some neurological diseases. A subset of calcium-binding proteins is designated as buffer proteins, because they are one component in maintaining Ca^2+^ homeostasis. Examples of these calcium buffer proteins are parvalbumin, calbindin-D9k, calretinin, and relevant to this study, calbindin-D28k (CB-D28k) [1]. A major role of CB-D28k is to protect cells from cellular destruction [4]. Early work in 1991 in hippocampal neuron cultures indicates that the level of CB-D28k, based on immunoreactivity, is directly related to reduction of free intracellular calcium concentrations and resistance of neurons to toxic effects [5]. Since then numerous studies have supported the neuroprotective role of CB-D28k in neural cells. Transduction or transfection of CB-D28k-virus vectors or plasmids enhance survival of neuronal cells to insults from hypoglycemia challenge [6], induction of toxicosis by calcium ionophores [7], amyloid beta-peptide [8], TNF-α-induced apoptosis [9], glutamate receptor antagonist (NMDA) [10], excitatory amino acids [7], [11], and ischemia [12]. One of these studies suggested that ‘fast’ Ca-buffers calretinin and CB-D28k, but not the ‘slow’ buffer parvalbumin (PV), protect neuroblastoma/retina hybrid cells from L-glutamate-induced cytotoxicity [10], which may emphasize the greater role of CB-D28k as a neuroprotectant as compared to PV and perhaps other buffer proteins.

Another mechanism in which CB-D28k might protect cells is by binding directly to caspase-3 and L-type calcium channel protein. In a cell-free system, CB-D28k inhibits recombinant caspase-3 enzyme activity [4]. This inhibition is probably due to CB-D28k binding to caspase-3 as determined with SDS-PAGE binding assays [4] and protein-capture chips [13]. CB-D28k also inhibits influx of Ca^+2^ via L-type calcium channel activity [14] possibly by binding to the L-type calcium channel protein [15], which could add to its cell protection properties.

Further evidence for the neuroprotective effects is the correlation of the lack of CB-D28k in spinal cord tissue with vulnerability to neuronal injury. A large subset of motor neurons in the spinal cord are especially vulnerable to destruction due to low cytosolic Ca^2+^ buffering [16]–[18], the presence of highly Ca^2+^-permeable AMPA receptors lacking the GluR2 receptor unit [19], [20], and unusual vulnerability to mitochondrial disruption [21]. Motor neurons lacking CB-D28k are readily damaged compared to motor neurons expressing CB-D28k [22]. Weak Ca^2+^ buffering capacity in a motor neuron is valuable under normal physiological conditions, because the buffer facilitates rapid relaxation times of calcium transients important for normal motor function. However, under pathological conditions, weak Ca^2+^ buffers are disadvantageous, because this condition accelerates a precarious circle of calcium dysregulation, mitochondrial disruption, and excitotoxic damage [23]. Consequently, motor neurons are especially vulnerable to damage during ischemia [24], amyotrophic lateral sclerosis [22], [23], and during infection with one virus examined, neuroadapted Sindbis virus [25].

Considering these published data on the role of calcium in viral replication generally and specific WNV replication, and putative CB-D28k neuroprotective properties, we conducted experiments to determine if CB-D28k in transfected cells inhibits WNV infection or cellular damage. In infected rodents, we determined that WNV immunoreactive (ir) cells were not co-localized with CB-D28k ir cells in the brain, suggesting that CB-D28k protective properties also occur *in vivo*.

## Results

**Figure 1:**
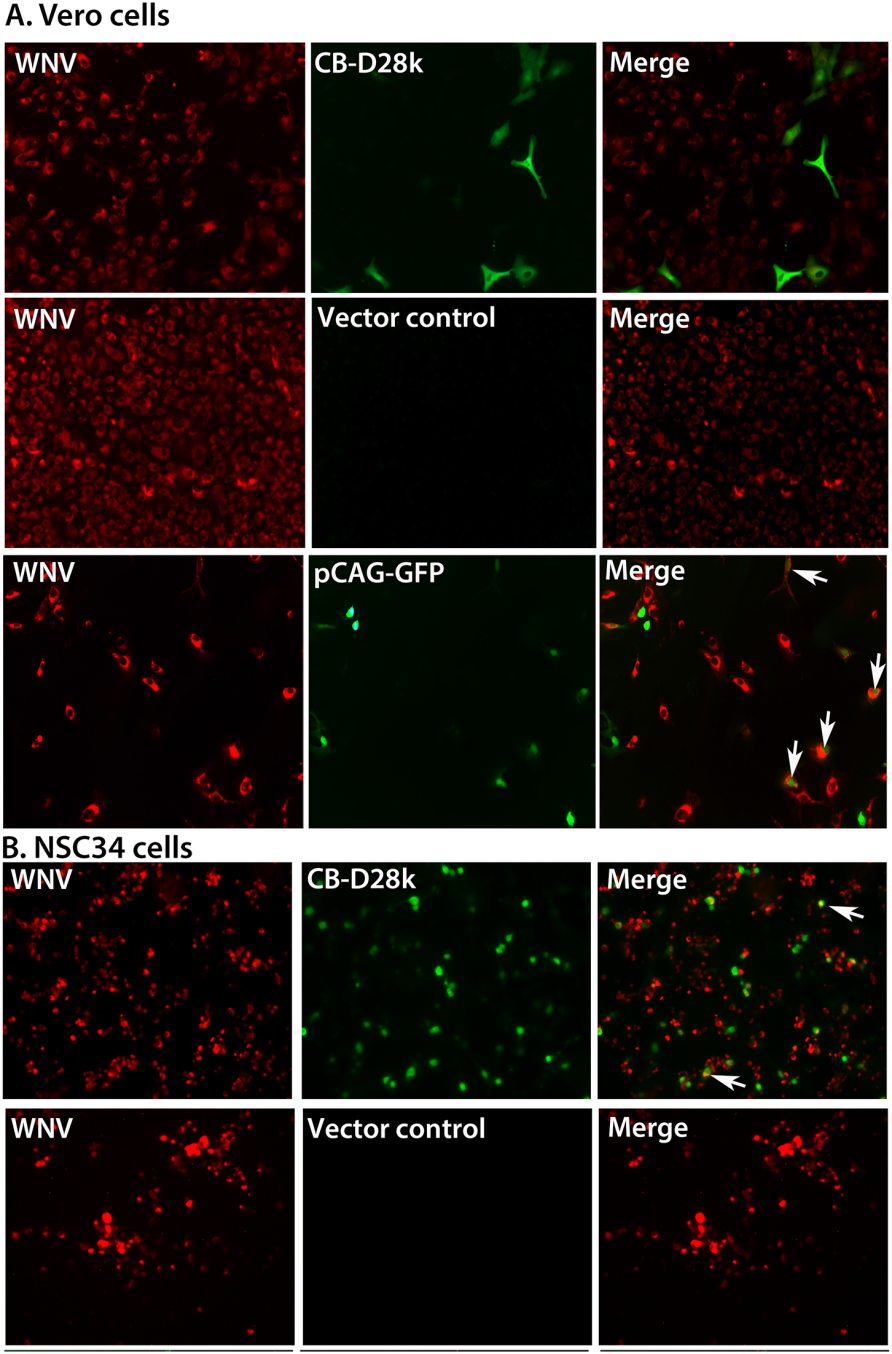
Reduced co-localization of WNV immunoreactivity (ir) with CB-D28k ir in (A) Vero 76 cells and (B) NSC34 cells transiently transfected with CB-D28k, vector control, or pGAG-GFP plasmids. Cells at 70–80% confluence were infected with a multiplicity of infection (MOI) of 5. Two days after viral challenge, cells on glass coverslips were fixed and immunostained. Arrows show co-localization of WNV with gene products of transfected plasmid. The transfection control was pCAG-GFP – CAG strong synthetic promoter for mammalian expression of green fluorescent protein (GFP).

The ability of CB-D28k to inhibit WNV ir was evaluated in Vero 76 and NSC34 motor neuron-like cells transiently and stably transfected with a vector expressing CB-D28k and with the empty vector control. An MOI of 5 in these experiments ensured that a large proportion of cells were exposed to WNV. Despite a high MOI, transiently transfected Vero cells with abundant CB-D28k ir were not co-localized with WNV ir (Figure 1A). Since many cells transfected with a control GFP-expressing plasmid (pCAG-GFP) were co-localized with WNV ir, the transfection process did not affect WNV replication. Likewise, large numbers of NSC34 motor neuron-like cells transiently transfected with the vector expressing CB-D28k were not co-localized with WNV ir (Figure 1B). Since these cells were infected with 5 MOI, CB-D28k-expressing cells appeared to be resistant to WNV replication. Nevertheless, there appeared to be occasional CB-D28k ir co-localized with WNV ir in NSC34 cells.

**Figure 2:**
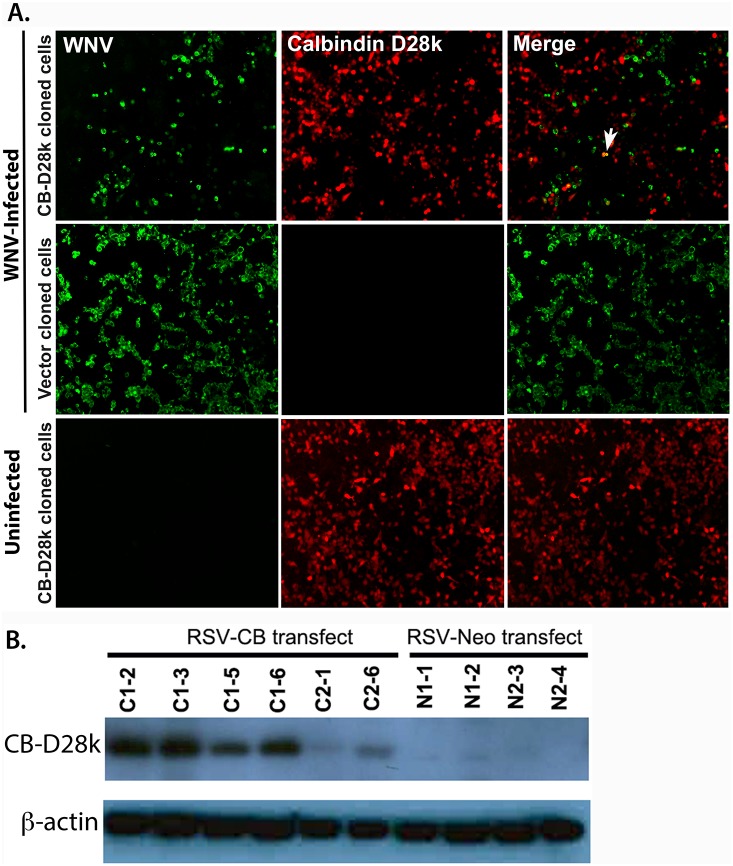
Reduced co-localization of WNV immunoreactivity (ir) with CB-D28k ir in NSC34 cells stably transfected with CB-D28k plasmid. (A) Cells stably transfected with RSV promoter–calbindin D28K/neomycin resistance (RSV-CB) or RSV promoter–neomycin resistance (RSV-Neo) plasmids [10] were infected with 5 multiplicity of infection (MOI) and immunostained 48 hours later. Arrow shows co-localization of WNV env ir with CB-D28k ir. (B) Western blot of CB-D28K in stably transfected NSC34 cloned cell lines. Clones C1–2 and N1–1 were used for WNV infection.

To support the observation that transient CB-D28k expression appeared to suppress replication of WNV, stably transfected NSC34 cells were generated and verified by western blot analysis (Figure 2B). The NSC34 cells were infected with 5 MOI of WNV. As observed in transiently transfected cells, large numbers of stably transfected NSC34 motor neuron-like cells expressing CB-D28k were not co-localized with WNV ir. Occasional CB-D28k ir cells co-localized with WNV ir cells (arrow) (Figure 2A). In contrast, many WNV ir cells were observed without presence of CB-D28k ir.

**Figure 3:**
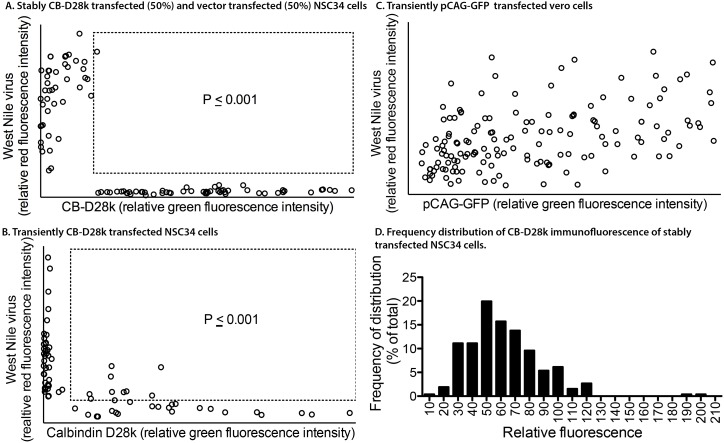
Quantitation of co-localization of CB-D28k ir with WNV env ir. (A) Stable transfection. Cell culture mixture of 50% CB-D28k stably transfected NSC34 cells and 50% vector control stably transfected NSC34 cells at 70–80% confluence were infected with 5 MOI and immunostained 48 hr later. The analyzed images were obtained from 5 representative areas of the slides using confocal fluorescent microscope. From those images, using ZEN software, based on the intensity of the staining, WNV-infected and CB-D28K transfected cells were quantified and plotted. The area within the dotted-line box represent cells where some level of co-localization of CB-D28k ir and WNV env ir was detectable. If CB-D28k did not affect viral replication, there would be no statistical difference between 50% of the number inside the box compared with 50% of the number outside the box. Therefore, chi square statistical analysis was performed by comparing the number of cells co-localized inside the box with the number of cells outside the box. (B) Transient transfection. NSC34 cells were transiently transfected with CB-D28k expression plasmid, and seeded at 70% confluence on glass coverslips. The next day the cells were infected with 5 MOI and analyzed by flow cytometry (total of 73 cells analyzed). Chi square statistical analysis was performed by conservatively assuming at an infection of 5 MOI that at least 60% of the CB-D28k ir cells would be infected by WNV, and co-localized with WNV env ir cells, and comparing the numbers inside and outside the box. P≤0.001. (C) Frequency of distribution of CB-D28k ir in stably transfected NSC34 cells. A total of 260 cells in fields of 100% stably transfected cells through three rounds of cloning were individually measured for relative ir and plotted on a frequency distribution histogram. Y-axis is represented as the percent (%) of total frequency.

To quantify the apparent resistance of CB-D28k-expressing cells to WNV replication, the intensity of the CB-D28k ir and WNV ir were quantified in individual stably transfected cells using confocal microscopy and ZEN blue software (Figure 3A). The experiment was performed with a mixture of 50% CB-D28k-stably transfected NSC34 cells and 50% vector control transfected NSC34 cells. The purpose of this mixture was to provide a 50% population of vector transfected cells expected to be infected by the virus, as compared to 50% of CB-D28k-transfected cells not expected to be infected by the virus. Co-localization of CB-D28k ir and WNV ir was identified with individual cell values within the boxed areas, as compared to the cell values outside of the boxed areas in Figure 3A. Very few cell values were within the boxed area, and were considered to co-localized with CB-D28k ir and WNV ir. The vast majority of the cells had WNV ir or CB-D28k ir, but not both. This quantitative analysis was also performed with transiently transfected NSC34 cells (Figure 3B). Similar results were obtained where most, but not all, CB-D28k ir cells were not co-localized with WNV ir. As a control, we quantified the relative intensity of WNV-positive cells and pCAG-GFP-positive cells in transiently transfected Vero cells. As expected, GFP expression of the control plasmid did not interfere with the production of WNV as evidenced by the distribution of co-localized WNV/GFP staining (Figure 3C). These data, therefore, support the ability of CB-D28k to suppress WNV replication.

**Figure 4:**
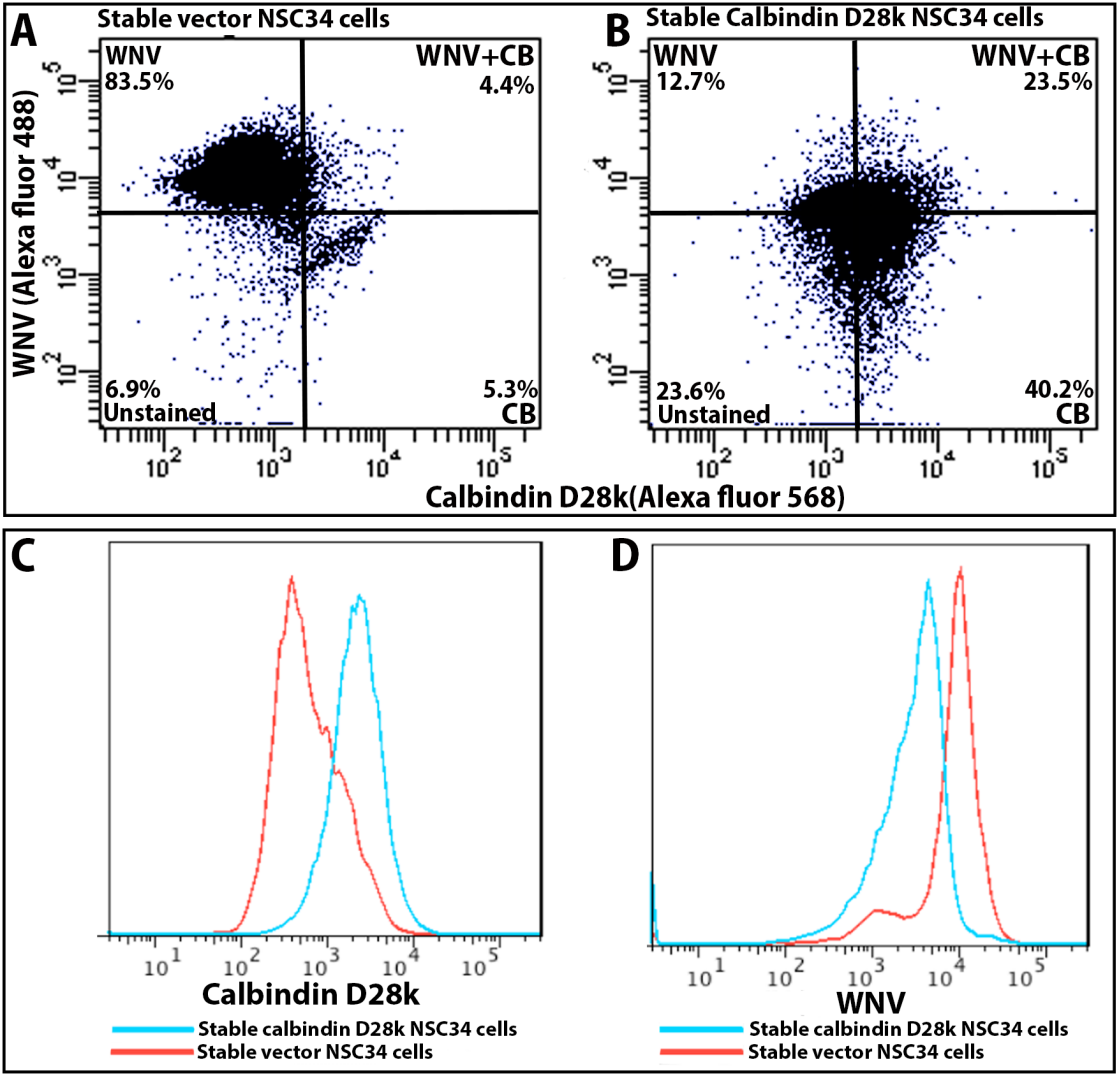
Reduced WNV replication in NSC34 cells expressing CB-D28k as detected by flow cytometry. NSC34 cells stably transfected with (A) the vector control plasmid or (B) CB-D28k plasmid were infected with WN02 at 5 MOI one day after seeding 6-well plates at 70–80% confluence. After 1-hour of adsorption, cells were washed and incubated for 48 hours. Cells were detached with 0.1% trypsin in MEM without FBS, and double-immunostained for WNV env (green-Alexa fluor 488) and CB-D28k (red-Alexa fluor 568). Flow cytometry analysis was performed with 10,000 cells for each group. NSC34 cells stably transfected with the vector control were 87.9% (83.5%+4.4%) immunoreactive for WNV env, as compared to 36.2% (12.7%+23.5%) with cells expressing CB-D28k. Histogram overlays reveal distinct peaks of CB-D28k-positive and –negative cells (C), and WNV-positive and -negative cells (D).

Flow cytometry was used to substantiate the same finding that CB-D28k-expressing NSC34 cells are resilient to WNV replication. NSC34 cells stably transfected with CB-D28k or the vector control were infected with WNV at 5 MOI. At 48 hours after infection, detached and fixed cells were double-immunostained for WNV env (green-Alexa fluor 488) and CB-D28k (red-Alexa fluor 568). Flow cytometry analysis was performed with 10,000 cells for each group (Figure 4). A large number (83.5%+4.4%  = 87.9%) of NSC34 cells stably transfected with the vector control were WNV env ir, as compared to 36.2% (12.7%+23.5%) WNV env ir with cells stably transfected with CB-D28k. The intensity of WNV ir in CB-D28k-transfected cells was substantially lower than the WNV ir intensity in the vector-transfected cells.

To investigate the effect of CB-D28k on viral replication, infectious viral titers were measured in the supernatants of stably or transiently transfected NSC34 or Vero cells at 18 or 24 hours after infection with WNV. The cells were infected with WNV at a MOI of 5, allowed to absorb for 1 hour, and washed with medium. As expected among individual transiently transfected cells, the level of CB-D28k expression, as identified by ir, was variable between cells. Unexpectedly, the level of CB-D28k expression was not uniform among individual stably transfected NSC34 cells, as illustrated in Figure 2. This was not due to mixed populations of stably transfected cells, because three rounds of cloning did not improve the uniformity of CB-D28k ir, as compared to CB-D28k ir from first-round cloned cells (data not shown). Figure 3C illustrates the variable intensity of CB-D28k ir. The relative immunofluorescence ranges from 200 to 10. The reason for this variability in cloned populations of cells is unknown, although a possible explanation may be that the level of differentiation amongst NSC34 motor neuron-like cells affects the amount of CB-D28k as observed in another study [22], and that individual cells in a culture may be at different stages of differentiation. Consequently, a large number of non-expressing CB-D28k ir cells were available in both transient or stably transfected cell cultures for the virus to infect and replicate. Five different experiments were performed to consistently demonstrate that CB-D28k reduced the amount of infectious WNV produced, although the reduction was relatively small compared to what the reduction might have been if the cells were uniformly expressing high amounts of CB-D28k (Table 1). The percentage of WNV reduction in transiently transfected NSC34 and Vero cells was 78% and 92% (Experiments 4 and 5). The percentage reduction of WNV titers in CB-D28k stably transfected NSC34 cells compared to control transfected cells (Experiments 1 to 3) ranged from 44% to 67%. A statistically significant difference (P≤0.05) was identified in data from experiment 2 due to larger numbers of replicates. Flow cytometry results substantiated these data. Assuming that the percentage reduction of WNV titers from stably CB-D28k-transfected cells (Table 1) were attributed to cells only expressing CB-D28k, the 40% of stably transfected NSC34 cells expressing CB-D28k as measured by flow cytometry (Figure 4) coincides with the percentage reduction of WNV titers (44–67%) in these cells.

**Table 1:** Effect of stable and transient CB-D28k transfection on WNV titers.

	Stable transfectedcloned cells^a^	MeanWNV titer^b^	Controltransfectedcloned cells^c^	MeanWNV titer	Percent reduction^d^(CB-D28k/control-transfected cells)
Expt^e^. 1. Stably transfectedNSC34 cells(24-hr harvest^g^)	Clone CB1/2	1.7×10^6^ (n = 3)^f^	Clone N1/2	7.7×10^5^ (n = 3)	
	Clone CB1/3	5.4×10^5^ (n = 3)	Clone N1/3	1.5×10^6^ (n = 3)	
	Clone CB1/6	5.4×10^5^ (n = 3)	Clone N1/6	1.7×10^6^ (n = 3)	
	Clone CB1/5	5.4×10^5^ (n = 3)			
	Clone CB2/1	3.2×10^5^ (n = 3)			
	Mean	7.3×10^5^	Mean	1.3×10^6^	44%
Expt. 2. Stably transfectedNSC34 cells(24-hr harvest)	Clone C1/1	7.0×10^5^ (n = 18)	Clone N1/1	2.0×10^6^ (n = 9)	65%*
Expt. 3. Stably transfectedNSC34 cells(18-hr harvest)	Clone C20	3.2×10^5^ (n = 2)	Clone N1/1	2.0×10^6^ (n = 2)	
	Clone C25	1.0×10^6^ (n = 2)			
	Mean	6.6×10^5^	Mean	2.0×10^6^	67%
Expt. 4. Transiently transfectedNSC34 cells(24-hr harvest)	–	1.7×10^7^ (n = 3)	–	7.7×10^7^ (n = 3)	78%
Expt. 5. Transientlytransfected Vero cells(24-hr harvest)	–	5.4×10^8^ (n = 3)	–	7.0×10^9^ (n = 3)	92%

*P≤0.05 t test.

a Transfected with RSV-CB-Neo vector.

b Serial dilutions of cell culture supernatant were added to indicator Vero 76 cells. Six days later cytopathic effect (CPE) was used to identify the end-point of infection [49], and 50% cell culture infectious doses (CCID50) per milliliter of cell culture supernatant.

c Transfected with RSV-Neo control vector.

d Percentage reduction of mean WNV titers from CB-D28k transfected cells compared to control transfected cells.

e Experiments 1 and 2 consisted of stably transfected NSC34 cells with one cycle of cloning. Experiments 3 consisted of stably transfected NSC34 cells with three cycles of cloning. Experiments 4 and 5 consisted of transiently transfected NSC34 and Vero cells, respectively.

f Number of replicates.

g Time of harvest after viral challenge.

**Figure 5:**
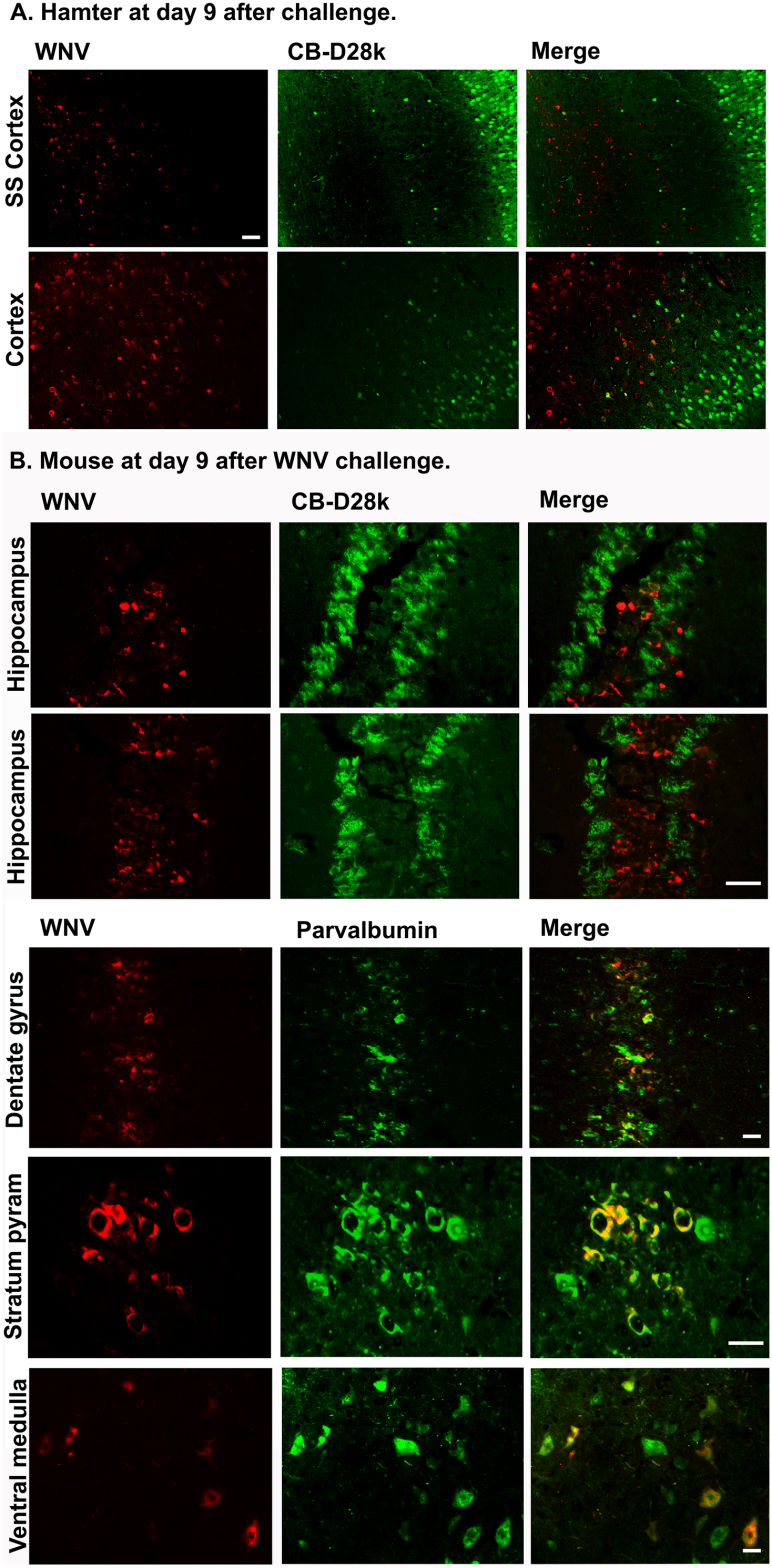
Absence of co-localization of CB-D28k ir with WNV env ir in (A) hamster, and (B) mouse neurological tissues. Rodents were infected s.c. with NY99 WNV. When WNV-infected animals could not right themselves, necropsy was performed, and immune-stained tissues were inspected for areas containing WNV env ir, plus CB-D28k ir or parvalbumin ir. SS Cortex: somatosensory cortex, Cortex: cerebral cortex, pyram: pyramidale. Bars = 50 µm.

To determine if the findings in cell culture had any relevance to WNV infection in rodents, we evaluated sections of neurological tissues from hamsters and mice subcutaneously infected with WNV. Necropsy was performed on a hamster and a mouse that could not right itself, and tissues were inspected for areas containing both WNV env ir, plus CB-D28k ir. Mouse tissues were also immunostained for parvalbumin (PV), a kinetically slow-acting buffer, as compared to CB-D28k, a kinetically fast-acting buffer [1], [11]. As observed in the cell culture studies, WNV env ir was not co-localized with CB-D28k ir in the somatosensory cortex and cerebral cortex of an infected WNV hamster (Figure 5A). Likewise, there was no co-localization in the hippocampus of an infected mouse (Figure 5B). Remarkably, PV ir was co-localized with WNV ir in areas found to have both markers, i.e., the dentate gyrus, stratum pyramidale, and ventral medulla.

**Figure 6:**
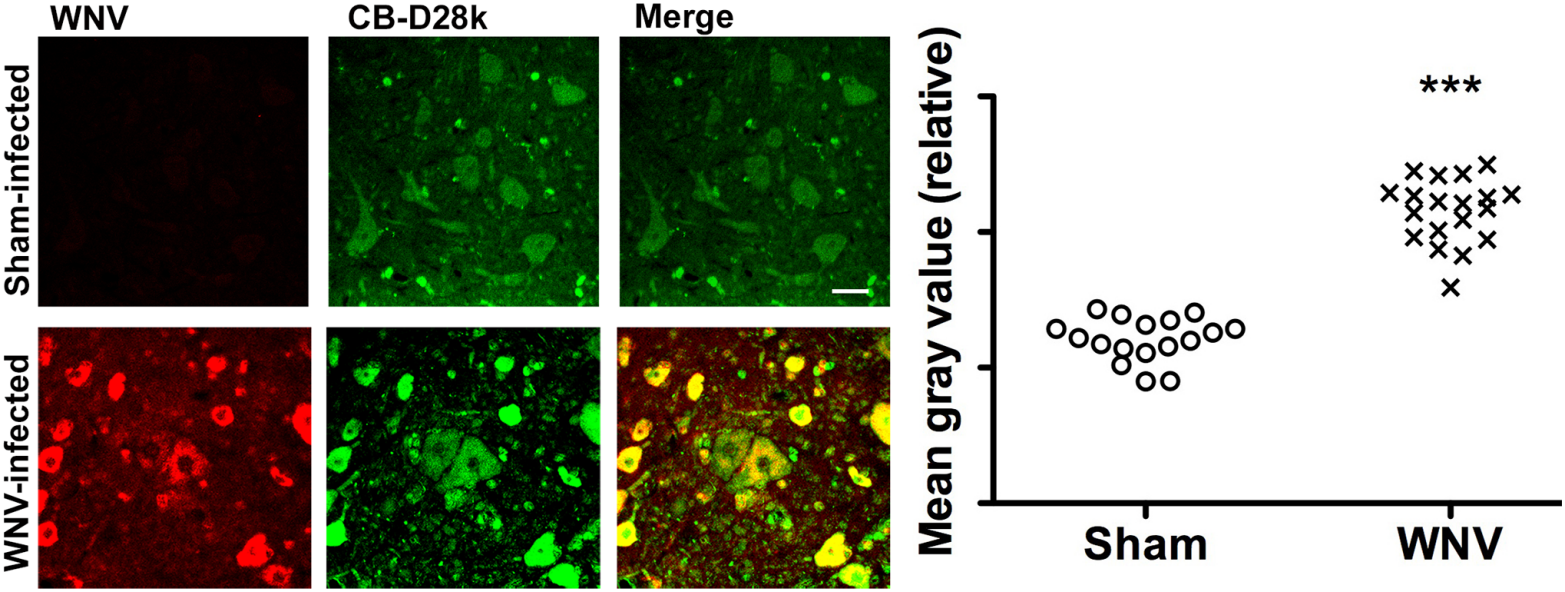
Induction of CB-D28k in spinal cord motor neurons by infection of hamsters by WNV. Hamsters were injected subcutaneously with NY99 WNV or sham (uninfected cell homogenate), and necropsied when WNV-infected animals could not right themselves. The cervical spinal cord was processed for immunohistochemistry (left). Intensity of CB-D28k was plotted from tissues in WNV- and sham-infected hamsters (right). Each dot represents individual cells from 4 different hamsters. ***P≤0.01 using t test.

WNV infects motor neurons to cause poliomyelitis-like disease in hamsters [26] and human subjects [27], [28]. These motor neurons do not express basal levels of CB-D28k [16], [18], [29] and have low calcium buffer capacities [30], which might contribute to their susceptibility to viral induced damage and replication. Since CB-D28k expression can be induced in response to other cellular insults [24], we tested the hypothesis that WNV infection can stimulate CB-D28k in surviving motor neurons in the spinal cord. Hamsters were injected subcutaneously with WNV or sham (uninfected cell homogenate), and necropsied when WNV-infected animals could not right themselves. The lumbar spinal cords were processed for immunohistochemistry, and the intensity of CB-D28k ir for each cell was plotted from tissues in WNV- and sham-infected hamsters (Figure 6). WNV infection statistically (P≤0.001) increased the degree of CB-D28k ir in ventral motor neurons as compared to those levels in neurons from sham-infected hamsters.

**Figure 7:**
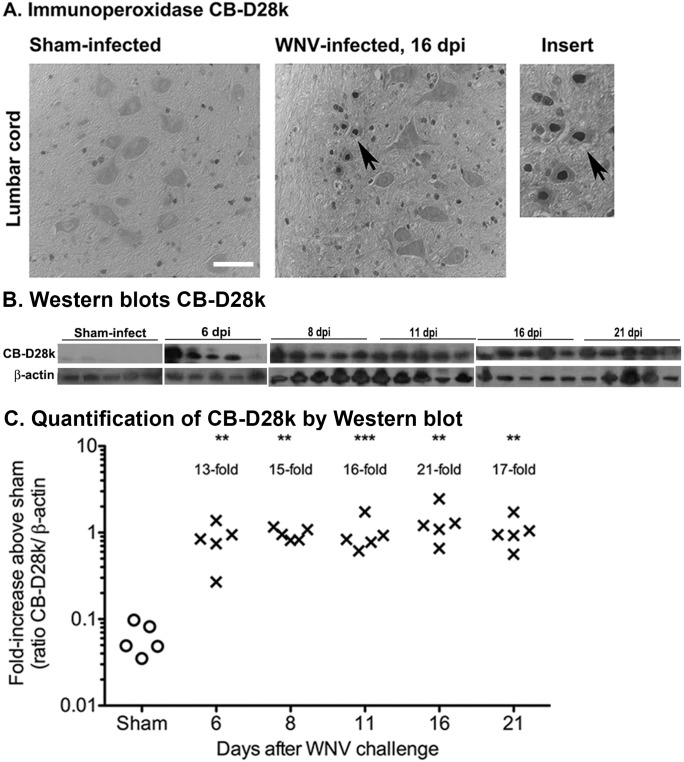
Induction of CB-D28K in the spinal cords of hamsters by infection with WNV. (A) Immunoperoxidase staining of CB-D28k in WNV- and sham-infected hamsters. Arrows: increased staining in motor neurons. (B) Hamsters were infected subcutaneously with WNV or sham. At 6, 8, 11, 16, and 21 days post-viral injection (dpi), subsets of hamsters were euthanized for collection of spinal cord tissues that were processed by western blot analysis for detection of CB-D28K. (C) Quantification of CB-D28k by western blot. Fold-increases were based on comparison to the sham-infected data. **P≤0.01, ***P≤0.001 compared to sham-infected data using ANOVA with Neuman-Keuls multiple comparison test.

To measure the CB-D28k induction over the course of the experiment, hamsters were infected subcutaneously with WNV or sham. At 6, 8, 11, 16, and 21 days after viral challenge, spinal cord tissues of hamsters were processed for immunoperoxidase detection of WNV env (Figure 7A), and western blot analysis for detection of CB-D28K (Figure 7B). As predicted, CB-D28k ir was not detected in motor neurons of the sham-infected hamsters. Beyond day 6 (data not shown) and more specifically at day 16 in Figure 7A, nuclear CB-D28k ir was detected in motor neurons. CB-D28k, as detected by western blot, was also induced beginning at the first day of measurement on day 6 and continued through day 21 (Figure 7B). CB-D28k normalized with β-actin expression was quantified by densitometry readings (Figure 7C). The fold-increase values ranged from 13- to 21-fold induction of CB-D28k between days 6 and 21 after viral challenge and compared to values from sham-infected hamsters.

## Discussion

The unique findings of this report were that WNV ir was markedly suppressed in Vero cells and NSC34 motor neuron-like cells transfected with CB-D28k and that CB-D28k inhibited virus replication. In hamster and mouse studies, we did not observe WNV env ir co-localized with CB-D28k ir in areas of the brainstem, limbic system, and cerebral cortex. In the spinal cord, WNV infects motor neurons [31]–[33], which do not express basal amounts of CB-D28k [18], [22], [23], [34]. When hamsters were challenged with WNV, the surviving motor neurons had induced the expression of CB-D28k. These and published data establish the framework of our hypothesis, which is that high calcium buffer-capacity of cells, as can be conferred by CB-D28k, protects neurons from WNV replication and WNV-induced damage. Also, spinal cord motor neurons without high buffer capacities are especially susceptible to WNV replication; however, the surviving neurons induce CB-D28k possibly as a neuroprotective response.

CB-D28k can theoretically affect WN neurological disease (WNND) by sequestering calcium to inhibit essential stages of viral replication as suggested by Scherbik and Brinton [3]. This current report established that CB-D28k can suppress WNV replication as observed with the lack of co-localization of WNV env ir and CB-D28k ir in Vero cells, and more relevantly to our neurological studies, in NSC34 cells having motor neuron-like properties [35]. Supporting these data is the observation from these studies that transfected CB-D28k reduces infectious virus titer, although by less than 1 log_10_ probably because of variable expression of CB-D28k in cloned cells.

Some of these CB-D28k beneficial effects might be relevant for protecting spinal cord motor neurons of WNV-infected rodents. CB-D28k-neuronal cells might be resilient to WNV replication, as suggested by a lack of co-localization of WNV env ir and CB-D28k ir in the brainstem, limbic system, and cerebral cortex, but a large subset of motor neurons in the spinal cord do not express basal levels of CB-D28k as described previously [16]–[18] and as shown in Figures 7A and 7B. The lack of CB-D28k expression in neurons might contribute to their exquisite susceptibility to WNV infection as observed in human and animal subjects [31]–[33], [36]. Surviving motor neurons, however, induce expression of CB-D28k in response to WNV infection, which might elicit neuroprotective cellular events to mitigate neuronal dysfunction and cell death. This may explain why WNV and CB-D28k ir can be co-localized in neurons in the spinal cord (Figure 6) and not in other neurons that express higher basal levels of CB-D28k (Figure 5) to protect them from WNV infection. The induction of CB-D28k in the ventral spinal cord also occurs in animals with ischemia [24]. Considering its known neuroprotective properties, induced CB-D28k in the WNV-infected spinal cord, and possibly other motor neuron diseases like ALS [23], might help protect neuronal cells from cellular damage.

Modeling the induction of CB-D28k by WNV in cultured motor neurons would verify observations in the spinal cords of infected mice and also facilitate mechanism studies. However, there are two current limitations. The first limitation is that the NSC34 neuronal cells divide and are not terminally differentiated. Consequently, the cells senesce in culture after 5–6 days. WNV-infected NSC34 neuronal cells die before they can induce CB-28k. As such, the cultured cells do not model motor neurons in the spinal cord. Fortunately, we have presumptively terminally differentiated NSC34 cells in differentiation medium. These differentiated NSC34 cells cease to proliferate, survive longer in culture, have increased size, and produce large neurite processes (data not shown). If these differentiated motor neurons are validated, future studies could address questions of induction of CB-D28k by WNV.

A study with the infection of borna disease virus (BDV), a neurotropic virus, in submucous and myenteric neurons of the colon offers further insights in the induction of CB-D28k during viral infection [37]. Fourteen weeks after viral challenge, 44% and 24% of the submucous and myenteric neurons, respectively, were BDV ir. Of the majority of BDV ir myenteric neurons immunoreactive to choline acetyltransferase (ChAT), a marker for motor neurons, CB-D28k ir was significantly elevated in BDV ir neurons as compared to ChAT ir neurons in control rats.

The co-localization of PV ir with WNV env ir in cells within the dentate gyrus, stratum pyramidale, and ventral medulla, as compared to no co-localization of CB-D28k ir with WNV env ir, suggest that the anti-WNV effects of CB-D28k are not non-specific for all calcium buffer proteins. PV is a kinetically slow-acting buffer and has a high affinity for Mg^2+^, whereas CB-D28k is a kinetically fast-acting buffer [1], [11], which may contribute to differences between these to proteins in response to WNV infection. Further work would need to be done to determine if there is any biological consequence of co-expression of WNV env ir with PV ir.

The susceptibility and cellular damage by WNV in low-buffer capacity cells may not be unique compared to other diseases. Catecholaminergic neurons in the substantia nigra express low amounts of calcium-binding proteins and are very vulnerable in Parkinson’s disease in human tissues [22], [38] and animal models [39]. In amyotrophic lateral sclerosis (ALS), motor neurons lacking calcium buffer proteins are severely damaged, but other spinal cord neurons expressing CB-D28k or PV are resistant to damage [16], [22].

The data of this report suggest that CB-D28k may suppress WNV replication to provide neuroprotective benefits to motor neurons of the spinal cord if CB-D28k is rapidly induced ahead of spread of the virus. There might be opportunities to pharmacologically induce CB-D28k to provide therapeutic benefits, or to utilize the effector mediating the anti-WNV effect of CB-D28k to suppress virus replication. One such effector might be an antagonist to an L-type calcium ion channel, because CB-D28k has been shown to decrease L-type calcium channel activity and modulate intracellular calcium in response to K+ depolarization in specific cultured cells [14]. Another approach might involve vitamin D3. Calbindin genes (D28k and D9k) are transcriptionally regulated by vitamin D3 in the intestine and kidney to mediate calcium absorption, although induction of CB-D28k by vitamin D3 in the central nervous system (CNS) is yet to be verified [40]. Cell culture studies do suggest, however, that it might be possible to induce CB-D28k in the CNS. Treatment of differentiated hybrid motor neuron cells (VSC 4.1 cells) with the active form of vitamin D (1,25 dihydroxyvitamin D3, 1,25(OH)_2_D3) increases the expression of calbindin D28k [22]. Moreover, strong binding sites for 1,25(OH)_2_D3 and immunoreactivity for the antibodies to vitamin D3 receptor (VDR) have been demonstrated in the CNS, including motor neurons [41], [42]. In animal studies, injection of 1,25(OH)_2_D3 in the cerebral ventricles of rats reportedly induced CB-D28k ir in ventral motor neurons [22]. Further studies need to be conducted to repeat and confirm the ability of 1,25(OH)_2_D3 to induce CB-D28k in neurological tissues and specifically in motor neurons of the spinal cord, and ultimately if 1,25(OH)_2_D3 can protect against WNV infection through the induction of CB-D28k. Moreover, identification of effectors of the anti-WNV effect of CB-D28k might lead to other strategies for treatment of WNND.

## Materials and Methods

### Ethics Statement

This study was carried out in strict accordance with the recommendations in the Guide for the Care and Use of Laboratory Animals of the National Institutes of Health. The protocols were approved by the Committee on the Ethics of Animal Experiments of Utah State University (USU) (IACUC approval #2125 and #2131). All efforts were made to minimize suffering. The work was done in the AAALAC-accredited Laboratory Animal Research Center of USU.

### Animals and Viruses

Adult female Syrian golden hamsters or C57BL/6 mice (>7 weeks old, 90–110 g) were used (Charles River Laboratories). Numbered animals were randomize to groups with the use of a random-number generator. WNV was propagated in MA-104 cells. The stock was diluted in minimal essential medium (MEM) immediately prior to subcutaneous (s.c.) injection in the groin area [43]–[45]. Hamsters were injected with a volume of 0.1 mL containing 5.7×10^7^ pfu of a New York WNV (NY WNV) isolate from crow brain [46], [47]. Mice were injected with a volume of 0.1 mL containing 2.5×10^6^ pfu of a WN02 isolate designated as KERN 515 from Dr. Robert Tesh (Mosquito, 10/05/07, Kern County, CA, TVP 10799 BBRC lot # WNVKERN515-01, University of Texas Medical Branch Arbovirus Reference Collection). These two strains of WNV were used because of suitable mortality in the two rodent strains used and to demonstrate that the findings of this report are not rodent- or strain-specific.

Animals were monitored twice daily for morbidity. Rodents were humanely sacrificed by overdose of pentobartitol (150 mg/kg, IP) if they did not step forward if prodded, or if they did not right themselves when placed on their backs. Ketamine/xylazine anesthesia and buprenorphine (0.05 to 0.1 mg/kg, SC, BID) analgesia were used when spinal cord injections were performed.

Virus was assayed using an infectious cell-culture assay [48]. Serial dilutions of cell culture supernatant were added to Vero 76 cells. Six days later the cytopathic effect (CPE) was used to identify the end-point of infection [49]. Four replicates were used to calculate 50% cell culture infectious doses (CCID50) per milliliter of cell culture supernatant.

### Cells/Plasmids

A murine motor neuron-like cell line, NSC34 [35], obtained from CELLutions Biosystems Inc, (Ontario, Canada) was propagated as per the supplier’s instructions. Both NSC34 and Vero 76 cells were grown in DMEM (Hyclone, Logan, UT), 10% FBS and 1% penicillin/streptomycin (Life Technologies, Grand Island, NY). The plasmids CB-D28k and its vector control, RSV-CB-Neo and RSV-Neo vector were provided by Prof. Beat Schwaller (University of Fribourg, Switzerland) [10]. Plasmids were linearized using Sma I before the transfection protocols. pCAG-GFP – plasmid, employing a strong CAG synthetic promoter (Addgene, plasmid 11150, Cambridge, MA) was used as a positive transfection control for expressing green fluorescent protein (GFP).

### Transfection

Magnetofection Neuromag (OZ Biosciences, France) procedure was adopted per the manufacturer’s instructions for transient transfection. NSC34 and Vero cells were seeded onto glass coverslips in 24-well plates. To prepare for transfection, 1 µg of linearized RSV-Neo or RSV-CB-Neo plasmids was incubated with magnetic beads at room temperature for 20 minutes. When cells were 60–70% confluent, 100-µL plasmid-bead complex was laid over each well, and the plates were kept on top of the magnetic bar for 20 minutes in a CO_2_ incubator. After 48 hours of incubation, cells were infected with WN02 strain of WNV at 5 MOI. Two days after the infection, cells were fixed for immunocytochemistry with freshly prepared 4% paraformaldehyde for 20 minutes.

Electroporation protocol was used to generate stably transfected cells. NSC34 cells (10^5^ cells) were electroporated with 1 µg of linearized RSV-CB-Neo and RSV-Neo plasmids using a Nucleofector kit (Lonza Cologne GmbH, Germany V4XC-2024). Transfected NSC34 cells were selected with G418 (500 µg/mL) (Life Technologies, USA) for 14 days. Individual clones were trypsinized cells collected with cloning cylinders under the microscope, and transferred to 96-well plates for further cloning and expansion. Selected clones were verified to have elevated CB-D28k expression by western blot. Clones 1/1, 1/2, 1/3, 1/5, 1/6, 2/1 were obtained from one round of cloning; whereas, clones C20 and C25 were obtained after three rounds of cloning.

### Immunocytochemistry

NSC34 and Vero cells grown on cover slips were fixed in 4% paraformaldehyde for 20 minutes at room temperature. For paraffin tissue sections from the animal studies, slides were de-waxed and rehydrated before proceeding with antigen retrieval steps using a de-cloaking chamber [43]. The cells/slides were washed three times with PBS for 5 minutes each. After permeabilization with 0.2% Triton X-100 in PBS for 3 minutes and washing three times with PBS, the cells were blocked for 1 hour in a PBS solution containing 5% normal goat serum (Sigma, St. Loius, MO) and 0.2% Triton X-100. This was followed by overnight incubation at room temperature with mouse monoclonal anti-WNV and goat polyclonal anti-CB-D28k antibody. After washing three times in PBS, 5 minutes each, samples were incubated 2 hour at room temperature with secondary antibodies (Alexafluor 488-conjugated anti-goat or Alexa fluor 568 conjugated anti-rabbit antibodies) diluted 1∶500 in PBS. Coverslips with the cells were again washed three times in PBS before being mounted for fluorescent microscopic examination. Peroxidase immunohistochemistry was performed using the same primary antibodies. Secondary antibodies were conjugated with horseradish peroxidase. DAB peroxidase substrate kit (Vector labs, USA) was used to visualize the staining products. Appropriate controls were performed along with every experiment to ascertain the specificity of the staining pattern. For controls, primary antibodies or all antibodies were removed, negative tissue/cell controls were included, and two fluorochromes were used for the same antigen, such as Alexafluor 488 conjugated anti-goat secondary antibodies replaced with Alexafluor 568 conjugated anti-goat secondary antibody. Images were obtained using confocal laser scanning microscopy, LSM 710 using ZEN software (Zeiss, USA) and processed using Adobe Photoshop 6.0 (Adobe, CA).

### Peak Intensity Quantification

The mean intensity of WNV ir and CB-D28k ir were quantified using ZEN blue module software (Zeiss, USA). Using the histo option and freehand tools on the software, cells were identified individually based on the morphology to obtain the staining mean intensity for individual cells. Data were plotted as a scatter diagram for both stable and transient transfection experiments.

### Western Blot

Total cell lysates were obtained from WNV-infected hamster lumbosacral cord tissues at different points after the infection. Fresh tissues were lysed with MPER extraction buffer plus a cocktail of Halt protease inhibitors (Thermo Scientific, Rockford, IL). Protein concentrations were determined using a Nanodrop spectrophotometer, (Nanodrop ND1000 Spectrophotometer). For each condition, 10 µg of protein per lane were separated on 10–20% SDS-PAGE gel in an Invitrogen Xcell Surelock Electrophoresis Cell (Life Technologies, Grand Island, NY) and transferred to PVDF membranes (EMD Millipore, Bellerica, MA). The membranes were blocked for 1 hour at room temperature with 5% BSA in PBS, and subsequently incubated with the primary antibody against polyclonal CB-D28k (Sigma, St.Louis, MO) in 5% BSA overnight at 4°C with shaking. The membranes were washed three times with PBS for 10 minutes each. The membranes were incubated with anti-rabbit horseradish peroxidase secondary antibody (1∶2000) (Thermo Scientific, Rockford, IL) for 1–2 hours at room temperature, and then washed as above. Antibody–antigen complexes were visualized using the Pierce ECL2 Western detection kit (Thermo Scientific, Rockford, IL) and exposed to GeneMate Blue autoradiography film (Bioexpress, Kaysville, UT). Densitometrically, CB-D28k bands were normalized against their respective β-actin using ImageJ software (NIH, Bethesda, MD).

### Flow cytometry

NSC34 cells stably transfected with CB-D28k- and vector-plasmids were seeded onto 6 well plates. When cells reached 70–80% confluence, they were infected with the WN02 strain of WNV at an MOI of 5. Forty-eight hours later, cells were trypsinized, washed with cold PBS, and fixed with freshly prepared 2% paraformaldehyde for 20 minutes on ice. After washing with FACS buffer (0.5% BSA, and 0.05% sodium azide in PBS), the cells were permeabilized with 0.1% Triton X100 for 5 minutes. After washing, cells were incubated with mouse monoclonal anti-CB-D28k (Synaptic Systems, Gottingen, Germany) and humanized monoclonal anti-WNV (MGAWN1) (Macrogenics, Rockville, MD) [43], [50] for another 30 minutes on ice, followed by appropriate fluorescently labeled secondary antibodies (Life Technologies, Grand Island, NY) for another 30 minutes. Clones were thoroughly washed twice with cold PBS to remove excess or unbound secondary antibodies. Cells were scanned with a BD FACSAria II (BD Biosciences, San Jose, CA) using appropriate filters. Controls with omission of primary antibody, incubation with irrelevant secondary antibody, and negative control samples were always performed along with experimental samples.
